# GA-Mediated Disruption of RGA/BZR1 Complex Requires HSP90 to Promote Hypocotyl Elongation

**DOI:** 10.3390/ijms24010088

**Published:** 2022-12-21

**Authors:** Panagiota Konstantinia Plitsi, Despina Samakovli, Loukia Roka, Aggeliki Rampou, Konstantinos Panagiotopoulos, Konstantinos Koudounas, Ioannis Isaioglou, Kosmas Haralampidis, Stamatis Rigas, Polydefkis Hatzopoulos, Dimitra Milioni

**Affiliations:** 1 Biotechnology Department, Agricultural University of Athens, 11855 Athens, Greece; 2 Laboratory of Virology, Scientific Directorate of Phytopathology, Benaki Phytopathological Institute, 14561 Athens, Greece; 3 Biology Department, National and Kapodistrian University of Athens, 15701 Athens, Greece

**Keywords:** *Arabidopsis*, HSP90, BR pathway, GA pathway, BZR1, RGA, GAI, hypocotyl elongation

## Abstract

Circuitries of signaling pathways integrate distinct hormonal and environmental signals, and influence development in plants. While a crosstalk between brassinosteroid (BR) and gibberellin (GA) signaling pathways has recently been established, little is known about other components engaged in the integration of the two pathways. Here, we provide supporting evidence for the role of HSP90 (HEAT SHOCK PROTEIN 90) in regulating the interplay of the GA and BR signaling pathways to control hypocotyl elongation of etiolated seedlings in Arabidopsis. Both pharmacological and genetic depletion of HSP90 alter the expression of GA biosynthesis and catabolism genes. Major components of the GA pathway, like RGA (REPRESSOR of *ga1–3*) and GAI (GA-INSENSITIVE) DELLA proteins, have been identified as physically interacting with HSP90. Interestingly, GA-promoted DELLA degradation depends on the ATPase activity of HSP90, and inhibition of HSP90 function stabilizes the DELLA/BZR1 (BRASSINAZOLE-RESISTANT 1) complex, modifying the expression of downstream transcriptional targets. Our results collectively reveal that HSP90, through physical interactions with DELLA proteins and BZR1, modulates DELLA abundance and regulates the expression of BZR1-dependent transcriptional targets to promote plant growth.

## 1. Introduction

Perplexed hormonal signaling affects common sets of cellular activities and developmental processes, and concurrently circuitries of signaling pathways integrate multiple hormonal and environmental signals that radically influence developmental plasticity in plants [1]. Direct crosstalk between signaling pathways has recently been elucidated, highlighting the fundamental entanglement of distinct hormones to promote diverse biological processes [2,3,4]. Brassinosteroids (BR) and gibberellins (GA) are two intertwined signaling transduction pathways that affect a wide range of developmental processes [5]. Aberrant brassinosteroid (BR) or gibberellin (GA) signaling pathways result in a range of highly similar phenotypes, including de-etiolation in the dark, delayed flowering, and reduced cell elongation and seed germination [6,7,8,9].

GA-promoted growth engages the binding of the ligand to a cytoplasmic-localized GIBBERELLIN INSENSITIVE DWARF 1 (GID1) receptor [10]. The current model suggests that GA binding to GID1 leads to conformational changes and nuclear localization of the receptor and promotes the interaction of the activated GID1 with DELLA proteins [11]. *Arabidopsis thaliana* has five DELLA family members with redundant functions that act as transcriptional regulators belonging to the GRAS family [12,13]. GA-INSENSITIVE (GAI) and REPRESSOR of *ga1–3* (RGA) DELLA proteins are involved in hypocotyl and stem growth [14,15], while (RGA-LIKE 2) RGL2 inhibits seed germination [3]. DELLAs presumably restrict plant growth by causing transcriptional reprogramming, and GA induces plant growth by removing the inhibitory activity of DELLA proteins through a ubiquitin-proteasome-dependent pathway [16]. Recent studies have shown that additional post-translational modifications like phosphorylation also regulate DELLA abundance [17]. GA and BR signaling pathways converge at the level of transcriptional control, as DELLAs interact with BRI1-EMS SUPPRESSOR 1 (BES1) and BRASSINAZOLE-RESISTANT 1 (BZR1) and block the DNA-binding activity of these transcriptional regulators [2,18,19]. BR perception by the membrane receptor kinase BRASSINOSTEROID-INSENSITIVE1 (BRI1) [20,21,22] leads to the association of the receptor with the coreceptor BRI1-ASSOCIATED KINASE 1 (BAK1) and induces the BR cascade [23]. BR-INSENSITIVE 2 (BIN2) has a negative regulatory function in BR signaling by phosphorylating BES1 and BZR1 to inhibit their transcriptional activity [24,25]. When BR levels are elevated, BIN2 is inactivated, BES1 and BZR1 resume their activity in the nucleus and bind to the promoters of multiple genes to regulate their expression in a BR-dependent manner [26,27,28]. Despite recent advances in understanding the direct crosstalk between the BR and GA signaling pathways, the molecular entity that drives the trajectory entanglement remains elusive.

HEAT SHOCK PROTEIN 90 (HSP90) is an evolutionarily conserved molecular chaperone that is considered a critical hub in the control of numerous molecular networks as it entails the functional competence of an extensive repertoire of signaling proteins, including transcription factors [29,30,31,32]. In this context, HSP90 influences a diverse spectrum of processes including development, cellular homeostasis and organismal evolution, and aberrant HSP90 results in a plethora of phenotypes [33,34,35,36,37]. The activity of the HSP90 molecular chaperone is central in the formation of BRI1 and BAK1 heterocomplexes during BR activation [38], in sustaining BIN2 nuclear function [39] and in BR-mediated feedback control through HSP90’s interaction with BZR1 and BES1 [40,41,42]. Compromised HSP90 activity reshapes the interaction dynamics of HSP90 with its client proteins, leading to abnormal degradation or destabilization of the HSP90 interactors [43].

Herein, we demonstrate that HSP90 is a key component of the GA signaling pathway, facilitating the integration of both the GA and BR pathways. BR-dependent GA-promoted cell elongation depends on the ATPase activity of the molecular chaperone, highlighting the HSP90-dependent GA-promoted destruction of the RGA/BZR1 nuclear complex. The inhibition of GA biosynthesis has a dramatic effect on hypocotyl elongation of *hsp90* mutant seedlings. Taken together, the integration of BR and GA pathways in jointly regulating hypocotyl elongation relies on HSP90 function.

## 2. Results

### 2.1. GA-Induced Hypocotyl Elongation Is HSP90 Dependent

**Figure 1:**
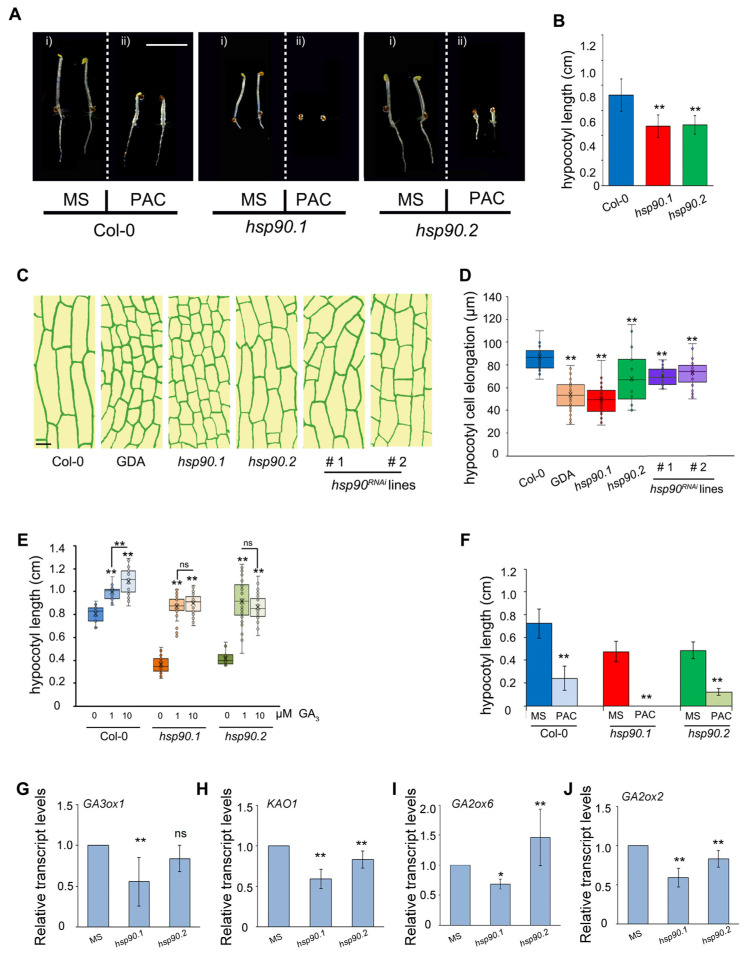
The *hsp90.1* and *hsp90.2* mutants respond differently to GA in the dark. (**A**) Hypocotyl phenotypes of 5-day-old wild-type (Col-0), *hsp90.1* and *hsp90.2* etiolated seedlings grown in MS (**i**) or in medium supplemented with 0.1 μM PAC (**ii**). Scale bars, 0.5 cm. (**B**) Hypocotyl length measurements of seedlings shown in (**Ai**). (**C**) Diagrammatic presentation of hypocotyl epidermal cells of 3-day-old etiolated seedlings of Col-0 grown on MS in the absence or presence of 2 μΜ GDA, and of *hsp90.1*, *hsp90.2* and of two independent *hsp90^RNAi^* lines grown on MS. Scale bars, 20 μM. (**D**) Quantitative analysis of hypocotyl epidermal cells lengths of the presented genotypes in (**C**). Box plots (**D**) display the first and third quartiles, split by the median; whiskers extend to include the max/min values. Data were analyzed by one-way analysis of variance (ANOVA) followed by Tukey’s test, ** *p* < 0.01. (**E**) Elevated concentrations of GA_3_ gradually restored the hypocotyl length of 5-day-old *hsp90.1* and *hsp90.2* etiolated seedlings. Box plots display the first and third quartiles, split by the median; whiskers extend to include the max/min values. Data were analyzed by one-way analysis of variance (ANOVA) followed by Tukey’s test, * *p* < 0.05, ** *p* < 0.01, ns, non-significant. (**F**) Quantification of hypocotyl elongation of 5-day-old etiolated seedlings grown in the conditions indicated in (**Aii**). (**G–J**). Relative expression analysis of genes involved in GAs biosynthesis (**G**,**H**) or catabolism (**I**,**J**) in 5-day-old Col-0, *hsp90.1* and *hsp90.2* etiolated seedlings. *GAPDH* was used as a reference gene. Data are means of three independent experiments and error bars indicate ± S.D. (*): *p* < 0.05, (**): *p* < 0.01, ns: non-significant according to *t*-test.

Increasing evidence supports the crucial role of HSP90 molecular chaperone in diverse aspects of plant development [30]. A seven-member gene family encodes the HSP90 protein in *Arabidopsis thaliana*. HSP90.1, .2, .3 and .4 isoforms localize primarily to the cytoplasm or the nucleus and share at least 85% identity at the amino acid level, suggesting functional redundancy [30]. To address the role of HSP90 in hypocotyl growth, genetic and pharmacological approaches were employed. Two Arabidopsis homozygous knockdown mutants *hsp90.1* and *hsp90.2* [31], when grown in the dark, showed similar de-etiolated phenotypes with significant reduction of hypocotyl elongation compared to the wild-type seedlings (Col) (Figure 1Ai,B). In line with these observations, two independent *hsp90^RNAi^* transgenic lines [44] also showed a significant reduction of hypocotyl elongation (Figure S1A). Τhe constitutively expressed *HSP90.2*, *HSP90.3* and *HSP90.4* were significantly reduced in *hsp90^RNAi^* transgenic lines, while the *HSP90.1* heat-inducible member was upregulated [44]. Interestingly, the application of HSP90 inhibitor geldanamycin (GDA) on wild-type seedlings phenocopied the hypocotyl length of the *hsp90* mutants **(**Figure S1B,C). To further expand our findings, we assessed epidermal cell length of 3-day-old etiolated hypocotyls of Col-0, *hsp90.1*, *hsp90.2*, *hsp90^RNAi#1^*, *hsp90^RNAi#2^* grown on MS and of Col-0 seedlings grown in the presence of 2 μM GDA. Transgenic seedlings grown on MS and wild-type seedlings grown in the presence of GDA showed significantly shorter epidermis cells compared to control (Figure 1C,D). These results indicate inhibition in cell elongation when HSP90 is pharmacologically or genetically compromised.

To determine whether *HSP90* genes are actively expressed during hypocotyl elongation, transgenic Arabidopsis lines *pHSP90.1::GUS* and *pHSP90.2::GUS,* harboring *HSP90.1* and *HSP90.2* promoters driving *β-glucuronidase* (*GUS)* gene, were employed. In 5-day-old dark-grown seedlings, GUS staining was mainly observed in the hypocotyl elongation zones (Figure S1D). Considering that genetic or pharmacological inhibition of HSP90 activity results in hypocotyl de-etiolation of dark grown seedlings, this suggests that in the dark HSP90 is involved in the hypocotyl developmental program. We also examined whether HSP90 function affects seed germination in the dark. Our results indicate that germination of wild-type (Col-0) Arabidopsis seeds and both *hsp90.1* and *hsp90.2* mutant seeds displayed no significant difference (Figure S1E).

In wild-type seedlings grown in the dark, response to gibberellin remains close to saturation [45,46]. To investigate how HSP90 function affects the hypocotyl growth in response to GA, we measured the hypocotyl length of 5-day-old wild-type, *hsp90.1* and *hsp90.2* etiolated seedlings grown in the presence of various concentrations of GA_3_. We found that both treatments with 1 or 10 μM GA_3_ significantly promoted hypocotyl elongation of Col-0 seedlings in a dose-dependent manner (Figure 1E and Figure S1F). Even though GA_3_ treatment of *hsp90* mutants restored the hypocotyl elongation phenotype, a dose-dependent response was not apparent, as application of higher GA_3_ concentrations did not lead to any further elongation of the hypocotyl in both *hsp90* mutants (Figure 1E and Figure S1F). These findings suggest a possible role of HSP90 in the GA signaling pathway that promotes hypocotyl elongation. 

To rigorously test this interpretation, we assessed the hypocotyl length in 5-day-old etiolated wild-type and *hsp90* seedlings after treatment with paclobutrazol (PAC), an inhibitor of GA biosynthesis. PAC application caused a significant decrease in hypocotyl elongation of wild-type and *hsp90.2* seedlings (Figure 1Aii,F). Interestingly, *hsp90.1* mutant showed extreme sensitivity to PAC treatment compared to the wild-type and *hsp90.2* seedlings, as a profound delay in germination was observed (Figure 1Aii,F). These findings show a differential response of the *hsp90.1* and *hsp90.2* mutants to GA biosynthesis inhibition, while the hypersensitive response of *hsp90.1* mutant to PAC further supports the view that HSP90 defects impair GA response or GA metabolism.

To further investigate whether the inhibition of hypocotyl elongation in *hsp90* mutants relies on GA metabolism, we examined the transcriptional profile of key genes involved in GA biosynthesis or catabolism in 5-day-old wild-type, *hsp90.1* and *hsp90.2* seedlings grown in the dark. The results showed that the transcriptional levels of *KAO1*, *GA3ox1*, *GA2ox2* and *GA2ox6* were significantly reduced in the *hsp90.1* mutant compared to wild-type seedlings (Figure 1G–J). However, in *hsp90.2* mutant, while both *KAO1* and *GA2ox2* transcript levels were down-regulated, the expression of *GA2ox6* or *GA3ox1* was induced or unaffected, respectively, when compared to the wild type (Figure 1G–J). The transcriptional response in the *hsp90.1* mutant shows an impaired GA biosynthesis/degradation pathway that can lead to highly defective homeostasis in the regulation of endogenous GA levels, as it can be deduced by non-germinating PAC treated *hsp90.1* mutant seeds after 5 days (Figure 1A,F). Taken together, our results suggest that HSP90 chaperone actively participates in the GA-mediated signaling pathway controlling the hypocotyl elongation and regulates GA catabolic/metabolic genes in the dark.

### 2.2. HSP90 Physically Interacts with DELLA Proteins

**Figure 2:**
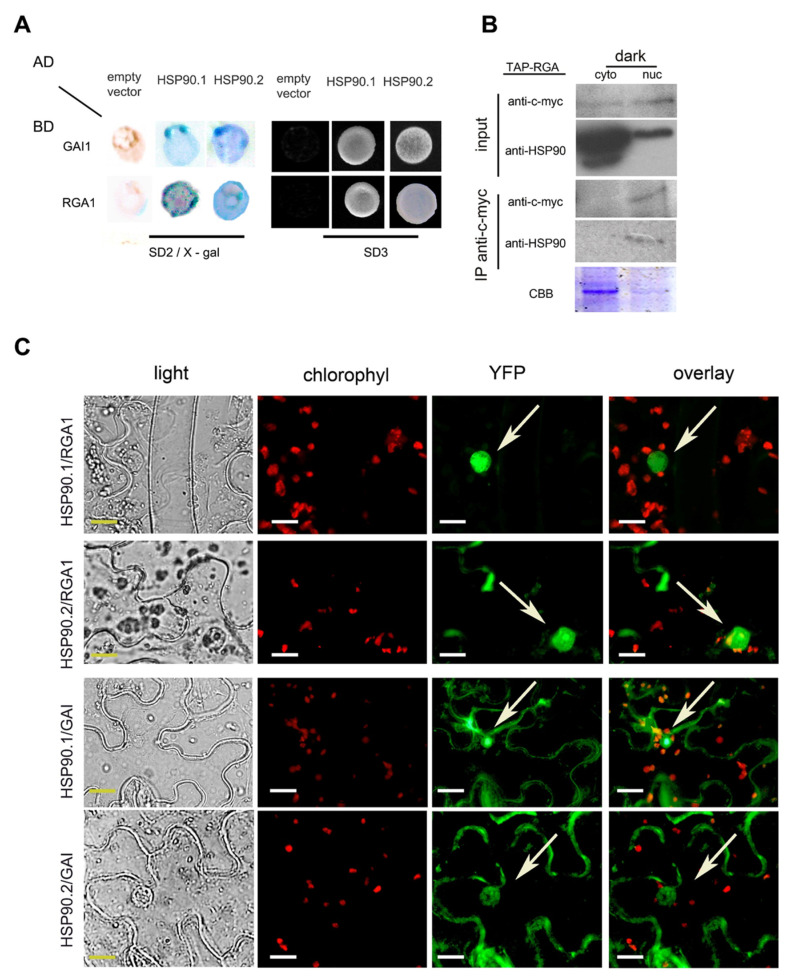
DELLA proteins, the negative regulators of GA pathway, are clients of the HSP90 molecular chaperone. (**A**) Yeast two-hybrid assays showing protein interactions between HSP90.1 or HSP90.2 and GAI or RGA, respectively. HSP90.1 or HSP90.2 were used as prey (AD), whereas GAI or RGA as bait (BD). Growth of co-transformants on SD^-Leu-Trp^ medium (SD2) confirmed the presence of both bait (BD) and prey (AD) plasmids were present in yeast cells. The positive interactions between HSP90.1 or HSP90.2 and RGA or GAI were also verified on SD2/X-gal and on SD-^Leu-Trp-His^ medium (SD3). (**B**) Co-immunoprecipitation assays showing interaction between HSP90 and RGA. in 6-day-old dark grown Arabidopsis seedlings. Coomassie brilliant blue (CBB) was used to evaluate protein levels in the cytoplasmic and nuclear fractions. (**C**) BiFC assays of HSP90.1- or HSP90.2-YFPc with GAI-nYFP or with RGA-nYFP in *N. benthamiana* leaf epidermal cells. Reconstituted fluorescent signal in nucleus and cytoplasm demonstrated protein–protein interactions. Arrows indicate the nuclei. Scale bars, 20 μm.

Since HSP90 is involved in the GA signaling pathway, we tested whether the molecular chaperone physically interacts with two members of the DELLA family, GAI and RGA, which are the main regulators of all GA responses in *Arabidopsis* [16,47]. Yeast two-hybrid assays showed that both HSP90.1 and HSP90.2 interacted with RGA and GAI proteins (Figure 2A). The physical interaction of HSP90 with RGA was further tested *in planta* by co-immunoprecipitation (co-IP) experiments. Proteins extracted from 6-day-old Arabidopsis *35S::TAP*-*RGA* transgenic seedlings [48] grown in the dark were separated into cytosolic and nuclear fractions. The abundance of RGA protein was higher in the nuclear fraction compared to the cytoplasmic one in contrast to the protein levels of HSP90 that were higher in the cytoplasm (Figure 2B and Figure S7). However, Western blots of the co-IP extracts using the appropriate antibodies revealed that the HSP90-RGA complex was prominent in the nucleus (Figure 2B and Figure S7). To further verify these interactions, we also performed bimolecular fluorescence complementation (BiFC) assays in tobacco cells, transiently expressing HSP90.1- or HSP90.2-YFPc and GAI- or RGA-nYFP. Fluorescence was observed in the nucleus and the cytoplasm of tobacco leaf epidermal cells indicating a physical interaction between HSP90 and DELLA proteins (Figure 2C). However, the interaction between HSP90.1 and RGA1 was mainly restricted to the nucleus when compared to HSP90.2 and RGA1 complex that was also apparent in the cytoplasm (Figure 2C). In epidermal cells co-expressing either HSP90.1- or HSP90.2-cYFP and the empty nYFP vector or the empty cYFP vector and GAI- or RGA-nYFP, no fluorescence signal was detected (Figure S2A). Negative and positive controls were also used to verify the specificity of interactions between HSP90 and DELLA proteins (Figure S2B,C). Our results show that HSP90 interacts with key components of the GA signaling pathway.

### 2.3. HSP90 Is Involved in GA-Mediated DELLA Degradation

It has previously been reported that hypocotyl elongation relies on cell expansion [49], which is restrained by the function of DELLAs [50]. The quintuple *della* mutant (q-*della*), lacking all five DELLA proteins, displays longer hypocotyl than wild type in the dark [48]. To clarify the functional interplay between HSP90 and DELLAs, the hypocotyl elongation of dark-grown q-*della* and wild-type seedlings was assessed in the presence of GDA. Interestingly, hypocotyls of q-*della* seedlings were less sensitive to HSP90 compromised function compared to GDA-treated wild-type hypocotyls (Figure S3A–C) indicating that perplexed signaling circuitries of different hormones affect the hypocotyl elongation of etiolated seedlings [2].

**Figure 3:**
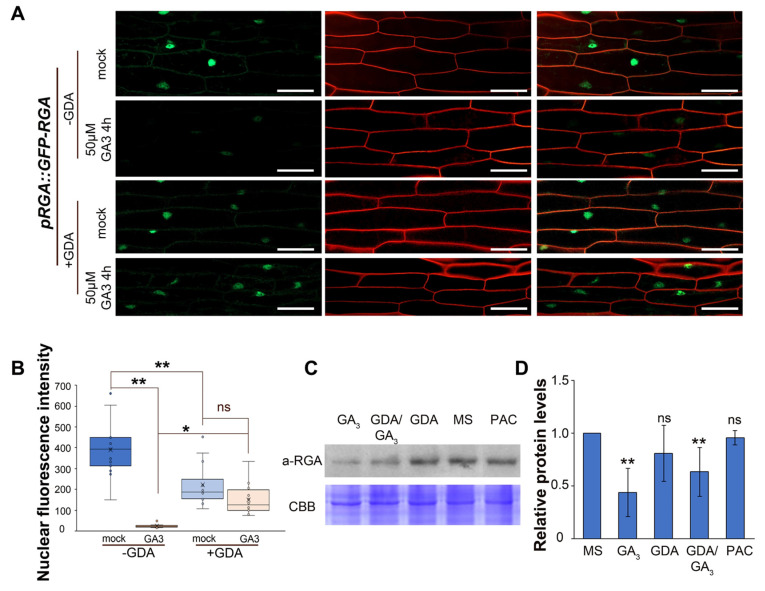
Pharmacological inhibition of HSP90 results in impaired GA response. (**A**) The reduction in GFP-RGA levels in response to GA_3_ treatment is dependent on HSP90 in hypocotyl cells close to the apical hook. Five-day-old etiolated *pRGA*::GFP-RGA seedlings were either untreated or treated with 50 μM GA_3_ for 4 h in the absence or presence of 2 μM GDA for 12 h. The cell patterns were visualized by using the cell wall stain propidium iodide (PI). Scale bars, 50 μm. (**B**) Quantification of the nuclear fluorescence intensity in hypocotyl cells of 5-day-old etiolated seedlings in the indicated treatments. To quantify the nuclear fluorescence, we assessed accordingly 26, 20, 24 and 28 hypocotyl cells. Box plots display the first and third quartiles, split by the median; whiskers extend to include the max/min values. Statistical analysis was performed using one-way analysis of variance (ANOVA) followed by Tukey’s test for multiple comparisons, ** *p* < 0.01, * *p* < 0.05, ns, non-significant. (**C**) Immunodetection of RGA protein levels in 5-day-old wild-type etiolated seedlings grown in MS or treated for 4 h with 25 μΜ GA_3_ or 2 μΜ GDA or 2 μΜ GDA and 25 μΜ GA_3_ or 0.1M PAC. Coomassie brilliant blue (CBB) was used for the evaluation of total protein levels. (**D**) Quantification of relative protein levels of RGA in 5-day-old etiolated seedlings. Data are means and error bars indicate ± SD. ** *p* < 0.01, * *p* < 0.05, ns, non-significant according to one-way analysis ANOVA followed by Tukey’s test for multiple comparisons.

Since HSP90 interacts with both RGA and GAI proteins (Figure 2), we investigated whether HSP90 modulates the stability of DELLA proteins. To address this, 5-day-old dark-grown seedlings expressing a *pRGA*::GFP-RGA translational fusion [51], were treated with GDA and/or GA_3_ (Figure 3A). In dark-grown hypocotyls, GA concentration has been reported to be lower in smaller cells of the apical hook compared to higher GA levels in longer cells of the elongation zone [52]. Live cell imaging showed that GFP-RGA fluorescence levels in the nuclei of smaller and still elongating cells just below the apical hook, were drastically reduced after GA_3_ treatment (Figure 3A,B). The fluorescence level of GFP-RGA was lower in the nuclei of hypocotyl cells of GDA-treated etiolated seedlings (Figure 3A,B). Interestingly, GA_3_ application in GFP-RGA-expressing seedlings pretreated with GDA did not cause any changes in the fluorescence intensity, suggesting that inhibition of HSP90 results in the stabilization of RGA in the nucleus (Figure 3A,B). In contrast, in already-elongated hypocotyl cells subjected to GA_3_, there was a decrease in GFP-RGA fluorescence in both GDA-treated and untreated seedlings (Figure S4A,B). Short and long hypocotyl cells responded differently to GA treatment upon HSP90 inhibition by GDA (Figure S4C). These results suggest that the pharmacological depletion of HSP90 hampers the GA-dependent RGA degradation in small hypocotyl epidermal cells located closer to the apical hook, supporting the view that the impact of HSP90 activity on hypocotyl growth depends largely on the developmental or differentiation stage of the hypocotyl cells.

Next, we examined whether HSP90 function regulates RGA protein levels in the whole Arabidopsis seedling by immunoblot analysis. RGA protein levels were strongly reduced after GA_3_ treatment, whereas no change in the endogenous RGA protein levels was detected under the effect of GDA or PAC (Figure 3C,D and Figure S7). Interestingly, co-treatment of seedlings at different time points with GDA and GA_3_ reduced the RGA protein levels less effectively than GA_3_ treatment alone, suggesting impaired GA response (Figure 3C,D and Figures S4D,E and S7). In conclusion, these results provide solid evidence that HSP90 function increases the efficiency of GA signaling by effectively promoting the degradation of RGA and controls the activity of the RGA regulator to modulate the downstream transcriptional output that mediates developmental responses.

### 2.4. HSP90 Modulates BR-Dependent GA-Promoted Cell Elongation 

**Figure 4:**
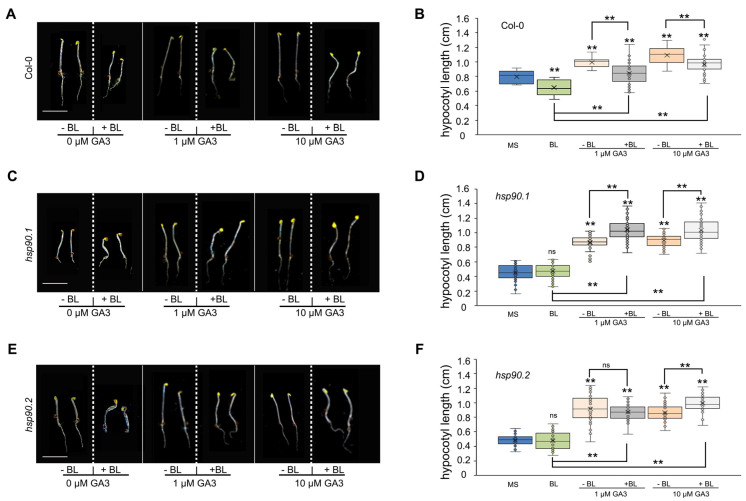
The interplay of GA and BR signaling pathways implicates HSP90. (**A**,**C**,**E**) Representative images of 5-day-old etiolated Col-0 (**A**), *hsp90.1* (**C**) and *hsp90.2* (**E**) seedlings growning on MS medium in the presence of 60 nM BL only or of GA_3_ increasing concentrations in the absence or presence of 60 nM BL. Scale bars 0.5 cm. (**B**,**D**,**F**) Hypocotyl lengths of Col-0 (**B**), *hsp90.1* (**D**) and *hsp90.2* (**F**) etiolated seedlings grown under the indicated conditions in (**A**,**C**,**E**). Number of measurements, n > 30. Data are means and error bars indicate ± SD. Box plots display the first and third quartiles, split by the median; whiskers extend to include the max/min values. Statistical analysis was performed using one-way ANOVA followed by Tukey’s test for multiple comparisons. (**): *p* < 0.01, ns: non-significant. Black asterisks indicate significant differences compared to Col-0.

HSP90 is involved in BR signaling by modulating the BIN2 translocation from the nuclear-cytosol trafficking in a BR-dependent mode [39], by assisting the compartmentalized cycle of BES1 [42], and by regulating the spatial distribution and accumulation of BRI1 and BAK1 receptors at the PM [38]. To clarify whether the HSP90 plays a role in the interplay between the GA and BR signaling pathways, we compared the response of hypocotyl elongation of *hsp90.1*, *hsp90.2* and wild-type etiolated seedlings treated with different concentrations of GA_3_ in the presence or the absence of BL (60 nM). Wild-type seedlings showed a significant reduction in hypocotyl length after BL application, whereas after GA_3_ treatment, they displayed a prominent increase in hypocotyl length (Figure 4A,B). A relevant increase, although at lower level, was also apparent in the GA_3_/BL-treated wild-type seedlings (Figure 4A,B). In both *hsp90* mutants, the application of BL did not cause any changes in the hypocotyl elongation suggesting that genetic depletion of HSP90 results in aberrant BR signaling (Figure 4C–F). Interestingly, we observed significantly increased hypocotyl elongation in *hsp90.1* etiolated seedlings when treated with both GA_3_ and BL in comparison to seedlings treated either with GA_3_ or BL (Figure 4C,D). *hsp90.2* etiolated seedlings showed no significant difference in hypocotyl length when treated with 1 μΜ GA_3_ in the presence of BL compared to seedlings treated solely with 1μM GA_3_ (Figure 4E,F). However, the application of 10 μM GA_3_ in the presence of BL significantly increased the hypocotyl elongation in *hsp90.2* etiolated seedlings in comparison to seedlings treated either with GA_3_ or BL. In conclusion, the hypocotyl elongation response of the etiolated *hsp90* mutants signifies the perplexed HSP90-mediated interplay of GA and BR signaling pathways. 

To further investigate the role of HSP90 in the convergence of the GA and BR signaling pathway regulating hypocotyl elongation in the dark, we measured the hypocotyl lengths of *hsp90* mutant and wild-type seedlings grown in the presence of brassinazole (BRZ), an inhibitor of BR biosynthesis. Although the GA_3_ application alleviated the inhibitory effect of BRZ and induced hypocotyl elongation in wild-type seedlings, it did not restore the hypocotyl length to the control levels (Figure S5A,B). Similarly, PAC application reduced hypocotyl length, while the combination of PAC with BL partially restored hypocotyl length (Figure S5A,B). Interestingly, deficiency of either GA or BL resulted in similar reduction of hypocotyl lengths as there were no significant differences in PAC or BRZ treated seedlings (Figure S5A,B), suggesting that both GA and BR pathways share equally important roles in the control of hypocotyl elongation of etiolated wild-type seedlings.

In *hsp90.1* seedlings treated with BRZ, we observed a reduction in hypocotyl elongation, which was restored to the mutant’s control levels in BRZ/GA_3_ treated plants (Figure S5C,D). Application of BL in PAC treated *hsp90.1* seedlings, recovered the delayed germination (Figure S5C,D). Our data indicate an HSP90.1 entanglement in both hormonal signaling pathways and that the seed germination is an HSP90.1-depedent developmental process that is prioritized via GA signaling. In *hsp90.2* seedlings, we observed that BRZ/GA_3_ application partially restored hypocotyl elongation caused by the deficiency of BR as in wild-type plants (Figure S5E,F). Furthermore, the combination of PAC and BL in *hsp90.2* seedlings did not alleviate the inhibitory effects on hypocotyl elongation caused by the PAC treatment (Figure S5E,F). The overall analysis of the *hsp90.1* or *hsp90.2* mutant response after GA/BL treatments as well as under deficiency of either BRs or GAs reveals a convergence between the two signaling pathways through the molecular chaperone.

### 2.5. The HSP90-BZR1 Complex Is Mainly Localized in the Nucleus

**Figure 5:**
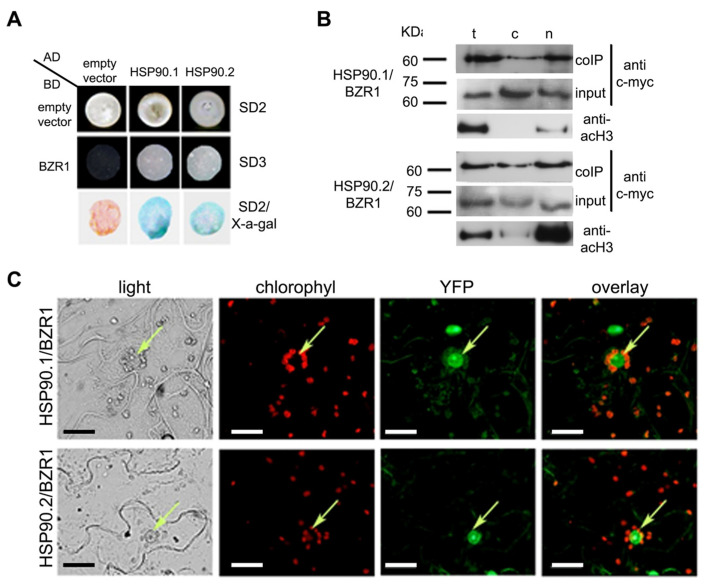
BZR1 interacts with HSP90.1 and HSP90.2. (**A**) Yeast two-hybrid assays using HSP90.1 or HSP90.2 as prey (AD) and BZR1 as bait (BD). Growth of yeast cells co-expressing the indicated construct combinations on SD^-Leu-Trp-His^ (SD3) or on SD2/X-gal media demonstrates interaction. (**B**) Co-immunoprecipitation assays of HSP90.1 or HSP90.2 with BZR1. Total (t), cytoplasmic (c) and nuclear (n) protein fractions were prepared from *N. benthamiana* leaves transiently co-expressing HSP90.1-HA or HSP90.2-HA with BZR1-c-Myc proteins, and immunoprecipitated with an anti-HA antibody. The immunoblots were probed with anti-c-Myc. For the evaluation of the fractionation the immunoblots were blotted with anti-histone H3 (anti-H3) antibody. (**C**) BIFC interactions assays of HSP90.1 or HSP90.2 and BZR1, in planta. Leaf epidermal cells of *N. benthamiana* were co-transformed with HSP90.1- or HSP90.2-YFPc and BZR1-nYFP constructs. Arrows indicate the nuclei. Scale bars, 20 μm.

In the absence of BR, active BIN2 phosphorylates and inactivates the BZR1 transcription factor [28]. Even though BZR1 has not been reported as being a client of the HSP90 molecular chaperone [40], co-IP assays showed that BZR1 interacts with HSP90 [41]. To rigorously test this discrepancy, we performed three sets of experiments. We first examined the protein–protein interactions in yeast two-hybrid assays and showed that both HSP90.1 and HSP90.2 strongly interacted with BZR1 (Figure 5A). Next, we performed co-IP assays with proteins extracted from cytoplasmic and nuclear fractions of tobacco protoplasts transiently co-expressing HSP90.1- or HSP90.2–HA–YFPc and BZR1–cMyc–nYFP. The interactions between BZR1 and the two members of the HSP90 protein family were detected in both the cytoplasmic and nuclear fractions (Figure 5B and Figure S7).

Furthermore, we examined the interactions in planta to substantiate the binding of HSP90 with the transcription factor BZR1. BiFC assays in tobacco leaf epidermal cells transiently expressing HSP90.1- or HSP90.2–HA–YFPc and BZR1-cMyc–nYFP confirmed the interaction between the molecular chaperone and BZR1 mainly in the nucleus (Figure 5C). Control tobacco leaves co-expressing the empty cYFP vector and ΒZR1-nYFP produced no YFP fluorescence signal (Figure S6). Our results resolved the discrepancy and corroborated previous data showing that BZR1 is a client of the molecular chaperone HSP90 [41]. 

### 2.6. Inhibition of HSP90 ATPase Activity Attenuates GA-Induced Destabilization of the RGA/BZR1 Complex

**Figure 6:**
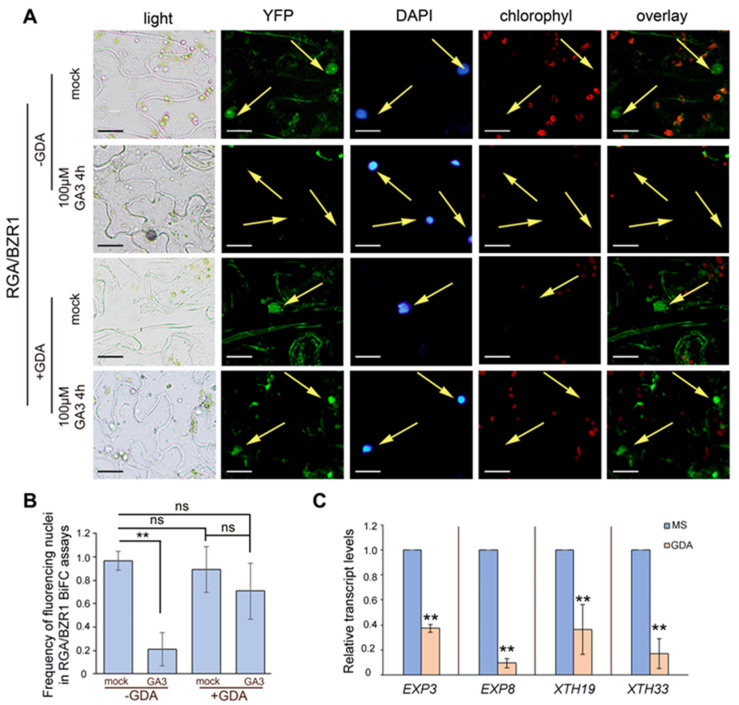
GA-promoted BZR1 transcriptional activity depends on HSP90-mediated RGA inactivation. (**A**) BiFC assays to test the interaction of RGA with BZR1 in *N. benthamiana* leaf epidermal cells. Tobacco leaves co-transformed with RGA-YFPc and BZR1-nYFP constructs were treated with 100 μΜ GA_3_ for 4 h or with 10 μΜ GDA for 12 h or with 10 μΜ GDA for 8 h followed by treatments with 100 μΜ GA_3_ for 4 h. Arrows indicate the nuclei. Scale bars, 20 μm. (**B**) Percentage of fluorescent nuclei after the indicated treatments in (**A**). Mean ± SD from at least 300 nuclei examined per treatment. (**): *p* < 0.01, ns—non-significant according to *t*-test. (**C**) Relative transcript levels of BES1/BZR1 target genes in 5-day-old etiolated seedlings of wild-type, *hsp90.1* and *hsp90.2* mutants under control conditions or in the presence of 2 μM GDA for 4 h. GAPDH was used as a reference gene. Data are means, and error bars indicate ± S.D. (**): *p* < 0.01, ns—non-significant according to *t*-test.

An outstanding crosstalk between BR and GA signaling pathways was observed, as DELLAs directly interacted with BR-activated BZR1 transcription regulator [2,18,53]. Since both DELLA and BZR1 are clients of HSP90, we studied the role of HSP90 in the GA/BR signaling trajectory and tested the recruitment of the ATPase activity of HSP90 in the formation of RGA/BZR1 complex. BiFC assays in tobacco leaves transiently co-expressing RGA-cYFP and BZR1-nYFP showed that RGA interacts with BZR1 in the nucleus (Figure 6A). When GA_3_ was applied, we observed a significant reduction in the frequency of fluorescent nuclei (Figure 6A,B). GA_3_ treatment, through the destabilization of DELLAs, abolished the interaction between RGA and BZR1 (Figure 6A) in line with previous results [2]. GDA treatment revealed a prominent fluorescent signal of RGA/BZR1 interaction in the nucleus, indicating that the association of RGA and BZR1 does not require the ATPase activity of HSP90 (Figure 6A,B). Interestingly, GA-promoted DELLA degradation was repressed in the presence of GDA as the interaction between RGA and BZR1 was apparent in a significantly higher number of nuclei of GDA/GA_3_ treated tobacco leaves compared to ones treated only with GA_3_ (Figure 6A,B), corroborating the observations of a delayed degradation process of RGA after GA_3_ application in seedlings pretreated with GDA. Driven by its own ATPase activity, HSP90 dynamically forms complexes with client proteins, sustaining a stable or stimulus-responsive conformation [54]. GDA binds to the unique N-terminal ATP-binding pocket of HSP90 and destructs the chaperone’s activity [55]. However, the notion that this HSP90-specific inhibitor promotes solely the degradation of client proteins, by loss of function can be misleading [56]. Previous results have shown that HSP90 activity is required for degradation of both membrane and cytosolic proteins [57,58], corroborating our findings that HSP90 mediates the GA-dependent RGA/BZR1 complex disruption.

DELLAs regulate BZR1 stability and activity thus affecting the expression of downstream transcriptional targets [2,18]. BZR1 and BES1 transcription factors have been previously shown to positively regulate the expression of the growth promoting *EXP8, XTH19* and *XTH33* genes [59,60], whereas DELLA proteins negatively regulate *EXP3* and *EXP8* expression [18]. Consistent with the hypocotyl length phenotypes, RT-qPCR analysis showed that treatments of wild-type seedlings with GDA remarkably decreased the transcript levels of *EXP3, EXP8, XTH19* and *XTH33* genes that are related to cell elongation when compared to the control seedlings (Figure 6C). These results support that HSP90 functional depletion reduces the expression of well-defined BES1/BZR1 transcriptional targets. Consequently, the HSP90 molecular chaperone by interacting with DELLA proteins, BES1 and BZR1 modulates the transcriptional activity of BZR1/BES1 factors and plays a key role in the BR-dependent GA-promoted cell elongation.

## 3. Discussion

A fundamental question in plant biology is how different endogenous cues affect common sets of cellular activities and developmental processes. Circuitries of signaling pathways that integrate multiple hormonal signals have recently been elucidated, highlighting that different hormones exert similar effects on diverse biological processes in plants [61,62,63]. BRs and GAs act on highly overlapping developmental responses [5] and the interplay between the two hormonal pathways is crucial for hypocotyl elongation [18,59].

Previous studies have shown that HSP90 is required for brassinosteroid, auxin and jasmonate hormonal responses in plants [38,39,44,64]. However, the involvement of HSP90 in the GA pathway has not been shown yet. Here, we provide evidence that HSP90 operates in the GA signaling cascade, since GA_3_ application partially rescued the impaired elongation phenotypes of *hsp90* mutants. Furthermore, the *hsp90* mutants displayed differential sensitivity to GA compared to wild-type etiolated seedlings, as hypocotyl length reached maximum elongation at low GA concentration. Optimal GA content has previously been reported to play major roles in the control of hypocotyl elongation and seed germination [65,66]. Our results suggest that HSP90 plays an important role in the GA homeostasis of etiolated seedlings, since HSP90 depletion caused alterations in the transcription levels of certain GA biosynthetic and catabolic genes. It is known that HSP90 acts as a master regulator of transcription, since a variety of transcription factors belong to its clientele [67]. Particularly, the altered expression of *GA3ox1* in *hsp90.1* mutant suggests that the final stage of GA biosynthesis to produce biologically active GA1 and GA4 [68] is decreased, supporting the view that HSP90.1 plays a major role in GA’s promotion of diverse developmental aspects such as seed germination and hypocotyl elongation. Additionally, the modulated expression of the *GA2ox2* gene in *hsp90* mutants indicates compromised regulation of the GA metabolic pathway.

The integration of the BR and GA pathways involves the direct interaction of protein regulator hubs like BZR1, BES1 and DELLAs [2,18,53], which all belong to the clients of HSP90. In the absence of GA, DELLA proteins accumulate and bind to the dephosphorylated active form of BZR1 and BES1, inhibiting their transcriptional activity, and therefore cell elongation. In the presence of GA, DELLA proteins are targeted for degradation through 26S proteasome, while GA induces the dephosphorylation of BZR1 and BES1 enhancing the expression of growth promoting genes [53]. Compromised HSP90 ATPase activity plays an important although cell-specific role in the nuclear accumulation of DELLA, while the cytoplasmic pool of DELLAs remains unaffected in the presence or absence of GA signaling. The activation and nuclear localization of BES1 or BZR1 depend on the BR-induced BIN2 inactivation and DELLA destabilization [2,18]. In its active form, BIN2 kinase phosphorylates and inactivates BZR1 and BES1 [24,69]. In their inactivated forms, the BR-responsive transcription factors BES1 and BZR1 are phosphorylated and mainly retained in the cytosol [70], where they are targeted for degradation by the 26S-proteasome [24]. Interestingly, it was recently demonstrated that high temperature activates BES1 through de-phosphorylation even in the absence of BRs [71].

**Figure 7:**
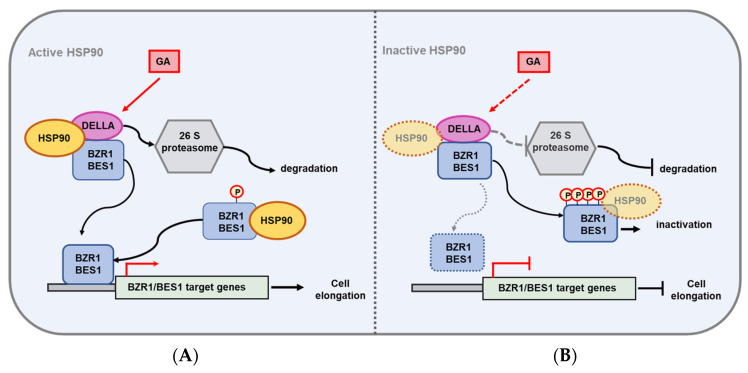
Schematic representation of the role of HSP90 in BL and GA crosstalk. The ATPase activity of HSP90 leads to degradation of DELLA proteins, allowing BR-dependent GA-promoted hypocotyl elongation and growth. (**A**) Active HSP90 interacts with partners of the BR signaling cascade, like BZR1/BES1 and of the GA signaling pathway, like DELLA proteins, controlling the phosphorylation state of BZR1/BES1 and facilitate the degradation of DELLA proteins to promote the expression of downstream transcriptional targets, which control the cell elongation of hypocotyl in the dark. (**B**) Inhibition of HSP90 function leads to aberrant BR and GA signaling caused by the accumulation of phosphorylated and inactivated BZR11/BES1 transcription factors, and the impaired degradation of DELLA proteins results in the arrest of hypocotyl elongation of etiolated seedlings.

Our study substantiates the role of HSP90 in the degradation of DELLAs and in the control of the transcriptional activity of BZR1, thus indicating that the molecular chaperone has a crucial role in the integration of distinctive signals driving plant growth and development (Figure 7). Given that HSP90 interacts with BZR1/BES1, controlling their phosphorylated/dephosphorylated state [41], herein we demonstrate that HSP90.1 and HSP90.2 directly interact with DELLA proteins. Both BZR1/HSP90 and RGA/HSP90 complexes are mainly localized in the nucleus. DELLAs are suggested to be related to the STAT proteins [72,73], and HSP90 is involved in the translocation of STAT3 from the cytosol to the nucleus mediating the downregulation of STAT3 transcriptional targets in mammalian systems [74].

While the inhibition of HSP90 activity did not disrupt the formation of the RGA/BZR1 complex, it compromised GA-induced DELLA degradation and blocked BZR1-induced gene expression. Therefore, the inhibition of HSP90 ATPase activity could reinforce the RGA/BZR1 heterodimer formation (levels) in deregulated GA signaling and homeostasis. GA-induced DELLA degradation derepresses the attenuated BZR1 activity which in turn regulates the expression of its target genes [2,18].

Since the nuclear pool of DELLA proteins, which depends on the HSP90 activity in a cell-specific manner, is of crucial importance in the formation of DELLA/BZR1 complexes, the active recruitment of the molecular chaperone is an essential element in GA signal responses. Our results indicate that HSP90 facilitates the function of its client proteins, possibly by modifying the levels of BZR1/DELLA complexes, thus modulating the balance between the GA and BR pathways in response to developmental cues. Interestingly, auxin and BR pathways control changes in the GA metabolism [75,76] and a crosstalk of GA, BR and auxin has been reported during cell elongation [77,78,79]. The function of HSP90 in the regulation of auxin signaling and distribution was recently revealed [44,64]. Therefore, we cannot exclude that the effect of HSP90 on GA signaling could also be mediated through auxin in addition to BR signaling.

The pivotal role of HSP90 in developmental networks relies on the concept that effective levels of HSP90 proteins should be available for signal transduction, defining the full activity of the network and the connectivity or the separation of the network components [80]. Genetic and protein interactions of HSP90 with client proteins in signaling pathways signify a concentration threshold [35,37]. Complete HSP90 depletion could lead to the fragmentation of the circuitry’s modules into isolated and unlinked subnetworks [81]. Major changes of the GA signaling dynamics are revealed when both the BR signaling pathway and the BR-GA crosstalk mechanism are disturbed [82]. GA levels were consistently decreased in *Arabidopsis* and rice mutants impaired in BR signaling [9,76]. Based on our data, we present a model (Figure 7) that predicts the existence of an HSP90-mediated link between two major hubs, DELLA and BZR1/BES1, which facilitates the integration of BR and GA hormonal signaling pathways and coordinate growth and development. HSP90 controls and adjusts the interplay of the BR and GA signaling pathways by forming physical complexes with RGA and BZR1, modulating the BZR1 activity, and influencing the abundance of DELLAs. The molecular chaperone facilitates the GA-induced degradation of DELLA proteins and affects RGA/BZR1 levels adjusting possibly the effective threshold level of complexes for normal growth. Given that HSP90, DELLA and BES1 levels are differentially modulated by heat stress [71,83,84], it would also be interesting to elucidate the functions of HSP90 in adjusting the dynamic relationship between gibberellins and brassinosteroid pathways under stress conditions. 

## 4. Materials and Methods

### 4.1. Plant Growth Conditions

*Arabidopsis thaliana* wild-type ecotypes Columbia-0 (Col-0), and Landsberg erecta (Ler-0), mutants and transgenic plants, and *Nicotiana benthamiana* L. (tobacco) plants were grown under 16:8 h, light:dark cycles at 22 °C. Arabidopsis seeds were germinated on Murashige–Skoog (MS) medium (Duchefa Biochemie B.V, Haarlem, The Netherlands) and Arabidopsis transgenic plants were selected on MS medium containing kanamycin (50 mg/L) (Merck, Darmstadt, Germany), or gentamicin (40 mg/L) (Merck, Darmstadt, Germany) or hygromycin (40 mg/L) (Merck, Darmstadt, Germany), under the same growth conditions. The T-DNA insertion lines SALK_007614 (*hsp90.1*, At5g52640) and SALK_038646 (*hsp90.2*, At5g56030) and the transgenic lines N16292 (*35S::*TAP-*RGAD17*), N16360 (*RGA::GFP-RGA*), N16298 (*q-della*) were obtained from the European Arabidopsis Stock Centre, Nottingham, UK. For hypocotyl length sensitivity assays, wild-type, transgenic and mutant plants were grown in the dark on vertical agar plates in plain MS medium or MS supplemented with different chemicals and concentrations as specified. Plates were photographed and the ImageJ software was used to measure the hypocotyl length of seedlings.

### 4.2. Phenotypic and Quantitative Analyses 

Hypocotyls of 5-day-old wild-type (Col-0, Ler-0) and different Arabidopsis mutant and transgenic seedlings were monitored under bright field microscope, and images were created and merged using Adobe Photoshop CS5 (version 9.01) software (Adobe, San Jose, CA, USA). Representative images of 3-day-old etiolated seedlings from each genotype, were printed and the shapes of the cells were drawn by hand. Then, the drawings were scanned, adjusting the contrast for clear images. The lengths of at least 30 hypocotyl epidermal cells per genotype were measured using the Image J 1.53v 21 software package (http://rsb.info.nih.gov/ij/ (accessed on 14 November 2022)). 

### 4.3. Yeast Two-Hybrid Assay

Interaction studies were performed in yeast strain Y2HGold using the Matchmaker GAL4 two-hybrid system according to the manufacturer’s protocol (Clontech, Mountain View, CA, USA). The full-length cDNAs of HSP90.1 and HSP90.2 were cloned into the pGADT7 vector (Clontech, Mountain View, CA, USA), and full-length BZR1, GAI and RGA were cloned into the pGBKT7 vector (Clontech, Mountain View, CA, USA). The primers used for genes amplification are presented in Supplementary Table S1. Yeast transformants were tested on Synthetic Dropout medium -Leu, -Trp (SD-2) and interactions were assayed on selective -Leu, -Trp, -His medium supplemented with 15 mM 3- amino-1, 2, 4-triazole (SD-3) (Sigma-Aldrich, St. Louis, MO, USA). To further test the specificity of the interactions, the β-galactosidase expression of the His+ colonies was analyzed by filter-lift assays using X-gal (5-bromo-4-chloro-3-indolyl-b-D-galactopyranoside).

### 4.4. Western Blot Analysis

Total protein was extracted from 5-day-old etiolated wild-type Arabidopsis seedlings. Plants were grown under control conditions (MS) or after treatment with different chemicals (25 μM GA, 2 μM GDA, 25 μM GA and 2 μM GDA, or 0.1 μM PAC) for 4, 6 or 12h. The plant samples were first ground in liquid nitrogen and then lysed in lysis buffer (50 mM Tris-HCl pH 7.5, 10% glycerol, 150 mM NaCl, 0.1% NP-40, 1 mM PMSF and 1x protease inhibitor cocktail). Equal amounts of total protein were resolved on an 8% SDS polyacrylamide gel either stained with Coomassie Brilliant Blue (CBB) or transferred to PVDF membrane (BioRad, Hercules, CA, USA). The primary antibody used for immunoblotting was anti-RGA (Agrisera, Vännäs, Sweden). The membrane was then washed and incubated with horseradish peroxidase-conjugated goat anti-rabbit IgG (Santa Cruz Biotechnology, Santa Cruz, CA, USA). The chemiluminescence was detected by using the Western Blotting Luminol reagent (Santa Cruz Biotechnology, Santa Cruz, CA, USA).

### 4.5. Bimolecular Fluorescence Complementation (BiFC) and Fluorescent Microscopy

HSP90.1–HA–YFPc, and HSP90.2-HA-YFPc [39], and BZR1–cMyc–nYFP, RGA-cMyc–nYFP, RGA-HA-YFPc and GAI-cMyc–nYFP were cloned either into the pSPYCE or the pSPYNE vector using appropriate primers (Table S1). BiFC assays were performed using Agrobacterium-mediated transformation into *Nicotiana benthamiana* leaves. At least three individual experiments were performed for each combination. BiFC protein–protein interactions were investigated using epifluorescence microscopy 3 to 4 d after infiltration [85]. YFP fluorescence was visualized using the fluorescent filter #41017, Endow GFP Bandpass Emission Filter (Chroma Technology Corp, Bellows Falls, VT, USA) and the chlorophyll autofluorescence was visualized with the UMSWG filter set (Olympus, Shinjuku City, NRT, Japan). Images were taken with an Olympus DP71 camera, using Cell^A (Olympus Soft Imaging Solutions). Final merging of images was performed using Adobe Photoshop CS5 (version 9.01) software (Adobe, San Jose, CA, USA).

### 4.6. Co-Immunoprecipitation Assays

Plant tissue from *35S*:TAP-RGA etiolated seedlings was harvested and ground in liquid nitrogen. Total protein extracts and nuclear or cytoplasmic fractions were prepared as described previously [39]. For HSP90/BZR1 co-immunoprecipitation assays, plant tissue from agroinfiltrated *N. benthamiana* leaf cells transiently and singly expressing or co-expressing the HSP90.1–HA–YFPc, HSP90.2-HA-YFPc and BZR1–cMyc–YFPn fusion proteins were used for protoplast preparation. Proteins were also co-immunoprecipitated from protoplast transiently expressing each construct, as controls. Total protein extracts were prepared from 2 mL of protoplast suspension, while nuclear and cytoplasmic proteins were fractionated from 6 ml protoplast suspension as previously described [39]. Protein extracts were incubated overnight with anti-hemagglutinin (anti-HA) antibody and then with Protein G PLUS-Agarose (Santa Cruz Biotechnology, Santa Cruz, CA, USA) at 4 °C for 4 h. For co-immunoprecipitation assays, the protein complex was precipitated and the corresponding protein extracts as inputs were resolved by SDS-polyacrylamide gel electrophoresis (SDS-PAGE) and detected by anti-c-Myc antibody (Santa Cruz Biotechnology, Santa Cruz, CA, USA), anti-HA antibody (Santa Cruz Biotechnology, Santa Cruz, CA, USA), anti-HSP90 antibody (Agrisera, Vännäs, Sweden) and anti-histone H3 antibody (Millipore, MA, USA). 

### 4.7. GUS Staining Assays

Histochemical beta-glucuronidase (GUS) activity assays were performed in different tissues of 30 independent *HSP90.1*::GUS [86] and *HSP90.2*::GUS [87] Arabidopsis Col-0 lines under control conditions. Quantitative GUS assays were carried out as previously described [86]. All measurements were repeated 3 times on 10 independently transformed plants from each line.

### 4.8. RNA Isolation and Expression Analysis

Total RNA was isolated from 5-day-old Col-0, hsp90.1 and hsp90.2 etiolated seedlings grown on MS medium, using the phenol-sodium dodecyl sulfate (SDS) extraction method. RNA concentrations were determined spectrophotometrically and verified by ethidium bromide staining on agarose gels. DNA was eliminated with RQ1 RNase-free DNase (Promega, Madison, WI, USA). Reverse transcription (RT) was performed on 3 μg of total DNA-free RNA using Superscript II Reverse Transcriptase (Invitrogen) according to the manufacturer’s instructions. PCR amplification for each transcript was performed using the pair of gene-specific primers. The linear PCR amplification of each transcript was confirmed in preliminary experiments by comparing the relative amounts of PCR products under low and high RT-PCR cycles of amplification. The gene specificity of RT-PCR products was confirmed by sequencing. qRT-PCR was performed by a PikoReal 96 Real-Time PCR System (Thermo Fisher Scientific, Waltham, MA, USA) using the SYBR Select Master Mix (Applied Biosystems, Waltham, MA, USA). Expression levels were calculated relative to GAPDH using the 2-ΔΔct method. Data are presented as average of three biological replicates, defined as independent plants of similar genotype/tissue, with error bars indicating standard deviation (SD). All qRT-PCR experiments were repeated at least three times with similar results. (*): *p* < 0.05 according to two-paired Students’ t-test and comparison to Col-0. Primers are designed according to the recommendations of Applied Biosystems and are listed in Supplementary Table S1.

### 4.9. Confocal Microscopy

GFP-RGA protein localization in hypocotyl cells of Arabidopsis was investigated in 3-day-old pRGA::GFP-RGA etiolated seedlings grown on MS medium. Living hypocotyls were stained with propidium iodide (PI; Thermo Fisher Scientific, Waltham, MA, USA) before confocal imaging. The hypocotyl images were captured using Zeiss LSM880 confocal microscope with Airyscan detector, under 488 nm laser excitation and 509 nm emission for GFP channel and 586 nm laser excitation and 600 nm emission for the Propidium Iodide (PI) channel.

## Data Availability

All relevant data can be found and are available within the manuscript and Supplementary Materials.

## Supplementary Materials

The supporting information can be downloaded at: https://www.mdpi.com/article/10.3390/ijms24010088/s1.
