# Supplementary material for: GA-Mediated Disruption of RGA/BZR1 Complex Requires HSP90 to Promote Hypocotyl Elongation
Source: Int J Mol Sci. 2022 Dec 21;24(1):88. doi: 10.3390/ijms24010088 (PMC9820706; doi:10.3390/ijms24010088)
Supplement: Supplementary file 1 [file ijms-24-00088-s001.zip › ijms-2067388-supplementary.pdf]

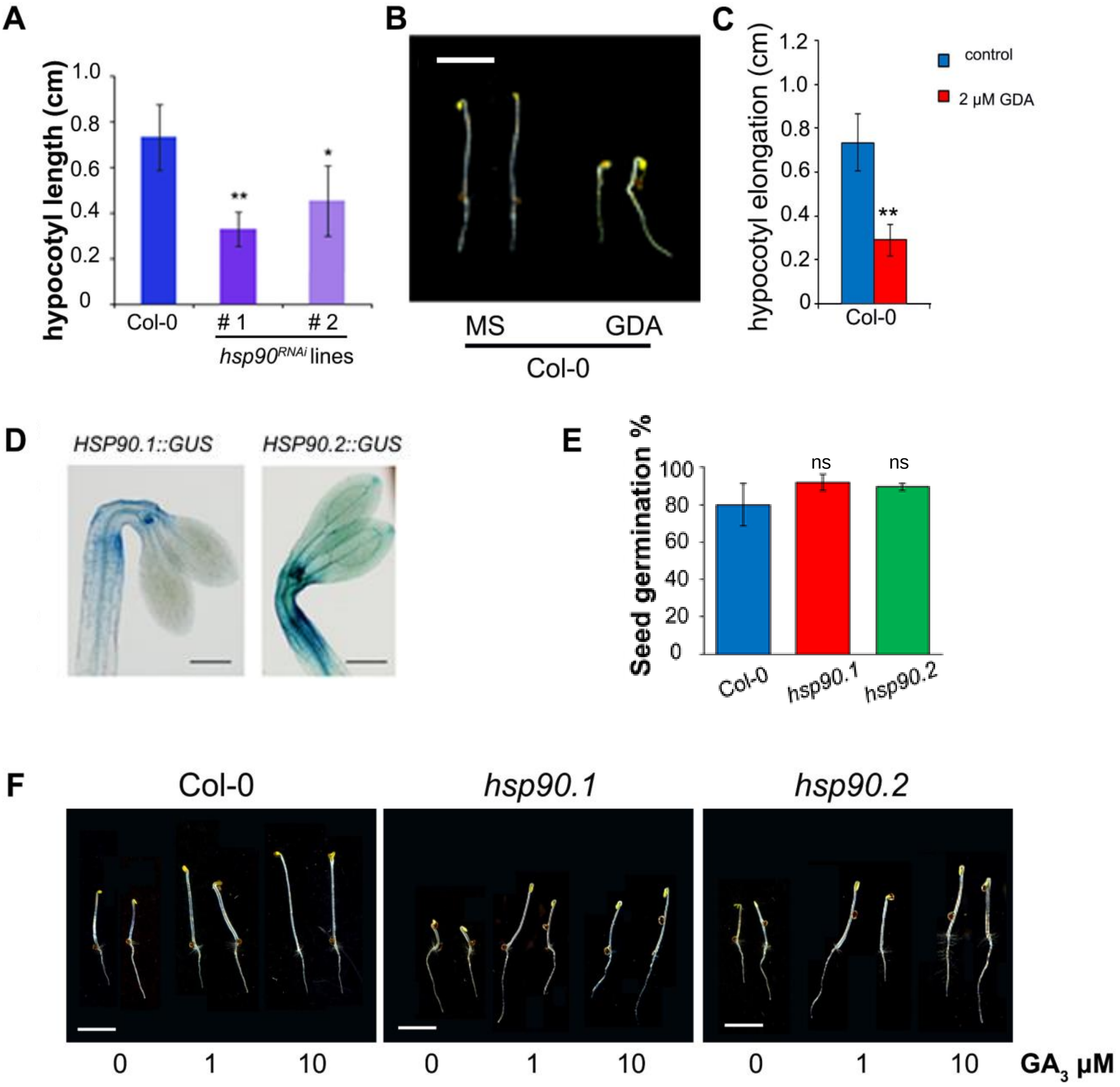

Figure S1. *HSP90.1* and *HSP90.2* genes are expressed in etiolated hypocotyls. **(A)** Hypocotyl length of 5-day-old etiolated seedlings of wild type (Col-0) and two independent *hsp90*^{RNAi} lines grown in MS medium. Number of measurements, n > 30. **(B)** The hypocotyl elongation of 5-day-old wild-type (Col-0) etiolated seedlings grown in medium supplemented with 2 µM GDA, was inhibited. Scale bars, 0.5 cm. **(C)** Hypocotyl length measurements of seedlings shown in (C). Number of measurements, n > 30. Data are means and error bars indicate ±SD. (*): p<0.05, (**): p<0.01, ns: non-significant according to two-tailed students' t-test. Black asterisks, comparison to Col-0. **(D)** *HSP90.1*::GUS and *HSP90.2*::GUS in the hypocotyls of 5-day old etiolated seedlings. Scale bars, 20 µm. **(E)** Percentages (%) of seed germination in the dark of Col-0, *hsp90.1* and *hsp90.2* are means (±SD) from three independent experiments (50 seeds per experiment). **(F)** Phenotypes of 5-day-old etiolated seedlings of *hsp90.1* and *hsp90.2* mutants grown in the presence of different concentrations of GA_3. Scale bars, 0.5 cm

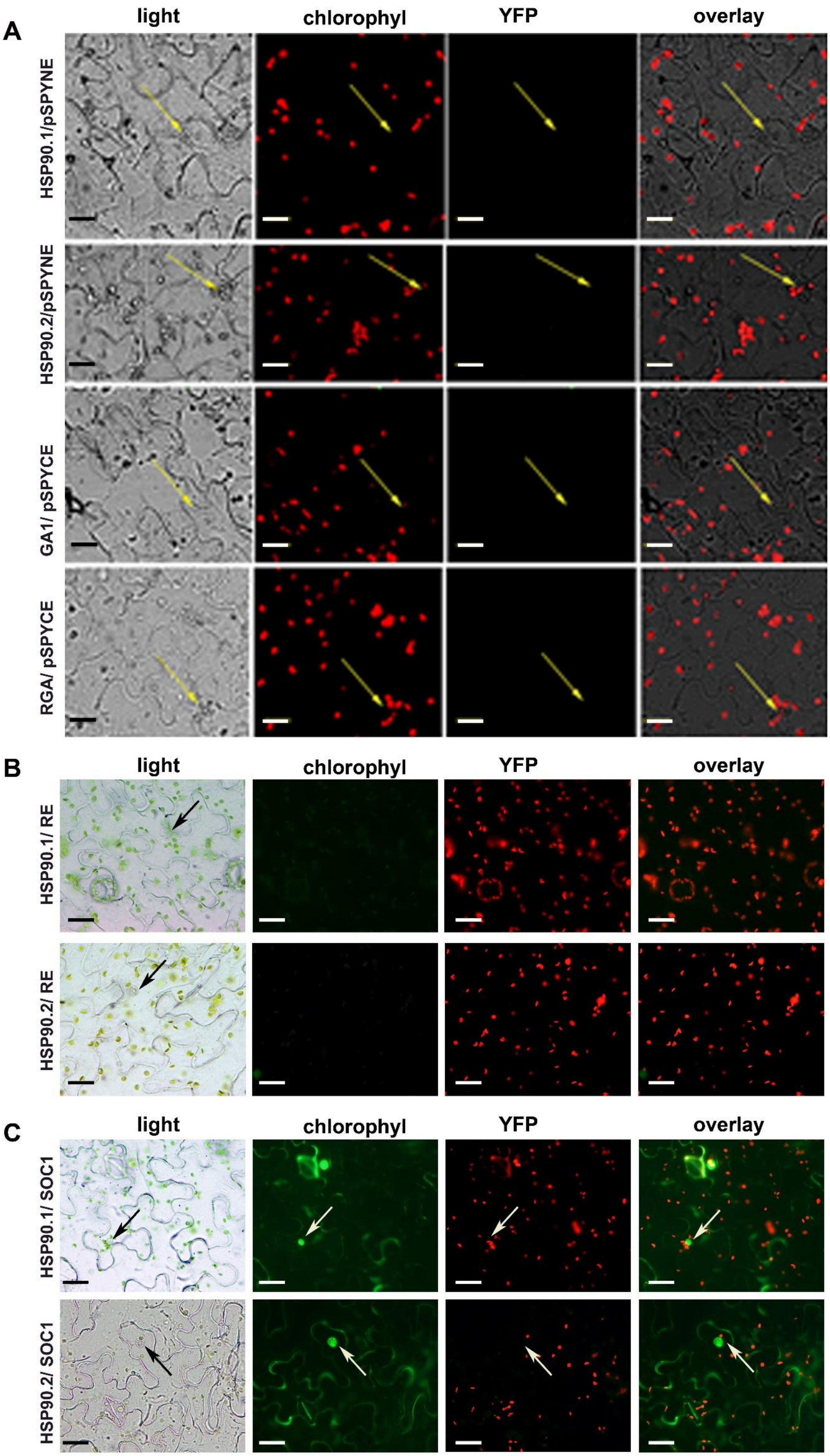

A
light
chlorophyl
YFP
overlay
HSP90.1/pSPYNE
HSP90.2/pSPYNE
GA1/ pSPYCE
RGA/ pSPYCE
B
HSP90.1/ RE
HSP90.2/ RE
C
HSP90.1/ SOC1
HSP90.2/ SOC1

Figure S2. Negative and positive controls of BiFC interaction assays of HSP90.1 and HSP90.2 with RGA or GAI. **(A)** *N. benthamiana* leaf epidermal cells co-transformed with HSP90.1- or HSP90.3-YFPc and pSPYNE-nYFP, or with pSPYCE-YFPc and GAI-nYFP or RGA-nYFP, were used as controls. No fluorescence was detected. **(B)** HSP90.1 and HSP90.2 do not interact with RABBIT EARS (RE) [**37**] , and therefore were introduced in *N. benthamiana* leaf epidermal cells as negative controls. No fluorescence was detected. **(C)** Both HSP90.1 and HSP90.2 interact with SUPPRESSOR OF OVEREXPRESSION OF CONSTANS1 (SOC1) [**37**], and therefore, were introduced in *N. benthamiana* leaf epidermal cells as positive controls. Reconstituted fluorescent signals in the nucleus and cytoplasm demonstrated protein-protein interactions in both cellular compartments. Arrows indicate the nuclei. Scale bars, 20 μm.

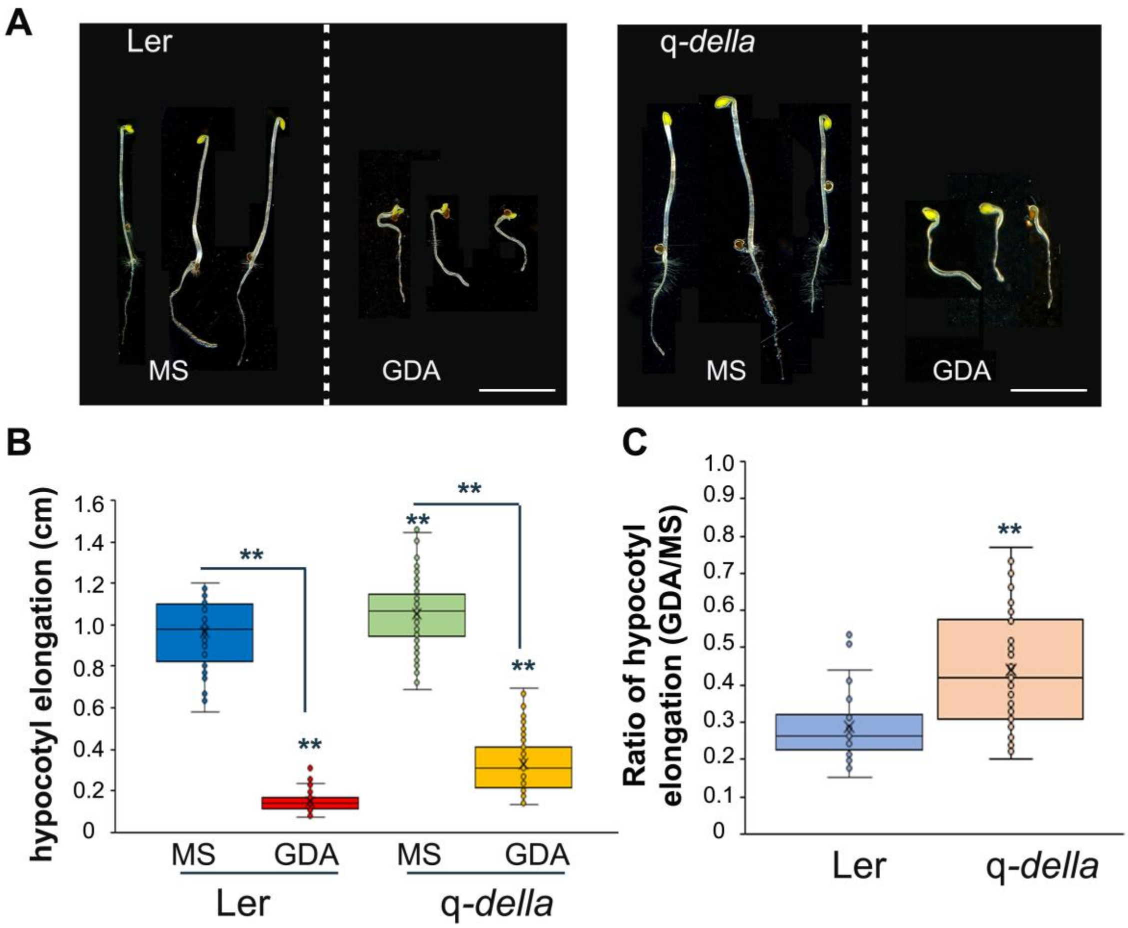

Figure S3. The hypocotyl growth of *q-della* mutants is partially insensitive to the inhibition of HSP90 function. **(A)** Hypocotyl images of 5-day-old wild type (Ler) and pentuple *della* etiolated seedlings grown in MS medium in the absence or presence of 2μM GDA. Scale bars, 0.5 cm. **(B)** Hypocotyl lengths of seedlings shown in (**A**). Data are presented as means and error bars indicate ±SD. Number of seedlings, n > 30. Data were analyzed with one-way ANOVA followed by Tukey's test, (**): $p<0.01$. **(C)** Hypocotyl length ratios (GDA/MS) of the quantified hypocotyl lengths in (B). Data are presented as means and error bars indicate ±SD. Number of measurements ≥ 30. Significant difference was compared to respective wild type. Student's t-test was performed (**): $p < 0.01$).

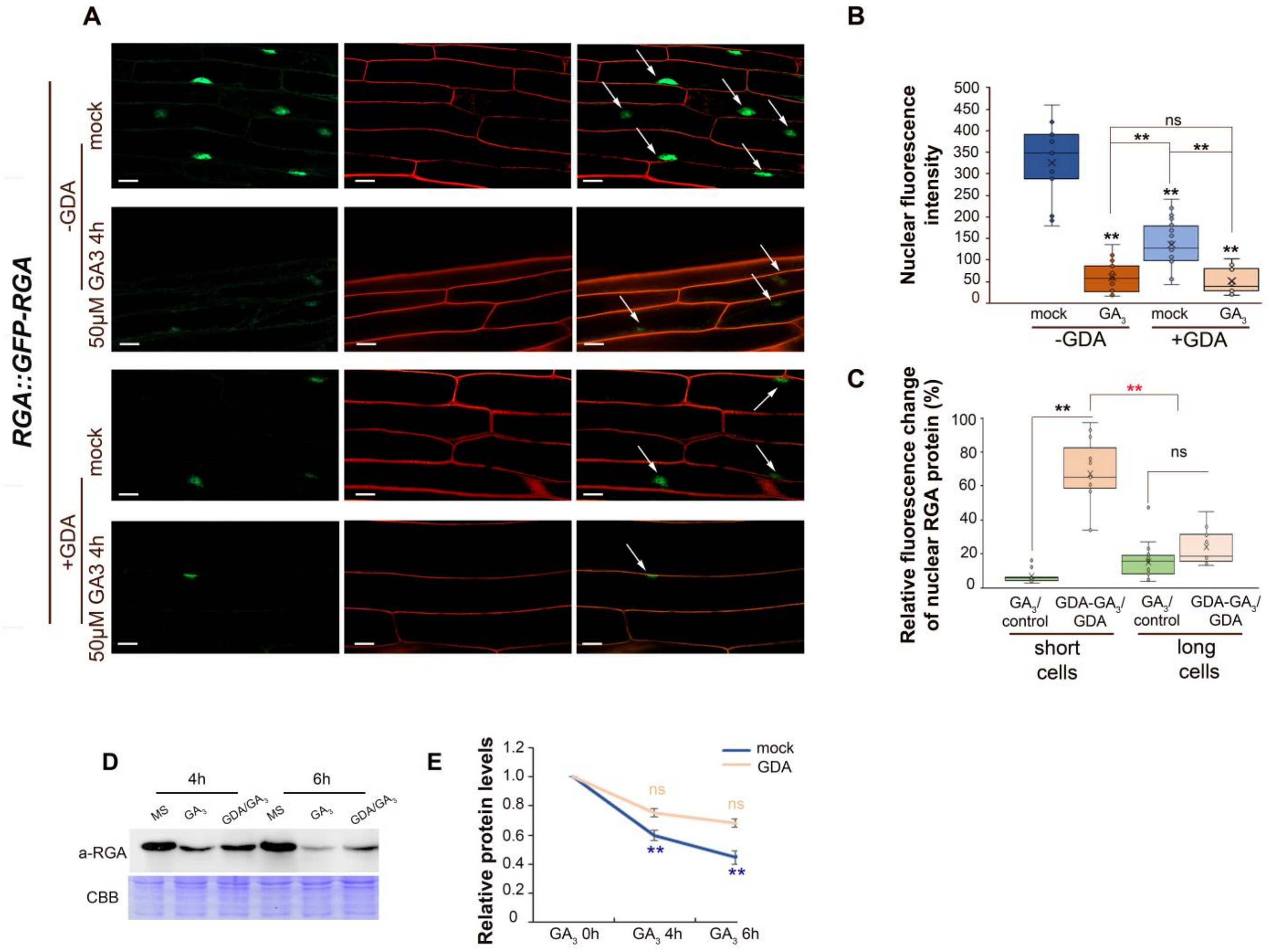

Figure S4. The effect of HSP90 pharmacological inhibition on RGA protein levels in the elongated hypocotyl cells of dark grown seedlings. **(A)** Fluorescent images of fully elongated hypocotyl cells showing GFP- RGA levels. 5-day-old etiolated *RGA:*:RGA-GFP seedlings with or without 50μM GA_3 treatment for 4h, in the absence or presence of 2μM GDA for 12h. The cell patterns were visualized by using PI. Scale bars, 50 μm. **(B)** Quantification of the nuclear fluorescence intensity in fully elongated hypocotyl cells of 5-day-old etiolated seedlings treated as indicated in (A). Box plots display the first and third quartiles, split by the median; whiskers extend to include the max/min values. Statistical analysis was performed using one-way analysis of variance (ANOVA) followed by Tukey test for multiple comparisons, **p<0.01. (**C**) Nuclear GFP-RGA fluorescence change (%) was assessed by the fluorescence ratios of short (**Figure 3B**) and long hypocotyl cells (**B**) under the indicated treatments, respectively. Statistical analysis was performed using two-way analysis (ANOVA) followed by Tukey test **(D)** Time course analysis of RGA protein levels in 5-day-old etiolated seedlings grown in control conditions and then treated with 25 μM GA_3 or 2 μM GDA and 25 μM GA_3 for the indicated time. Coomassie brilliant blue (CBB) was used for the evaluation of total protein levels. **(E)** Quantification of RGA relative protein levels after GA_3 application in the presence or absence of 2 μM GDA. Data are means and error bars indicate ±SD. **(**):** P<0.01, ns**:** non-significant according to one-way analysis ANOVA followed by Tukey for multiple comparison.

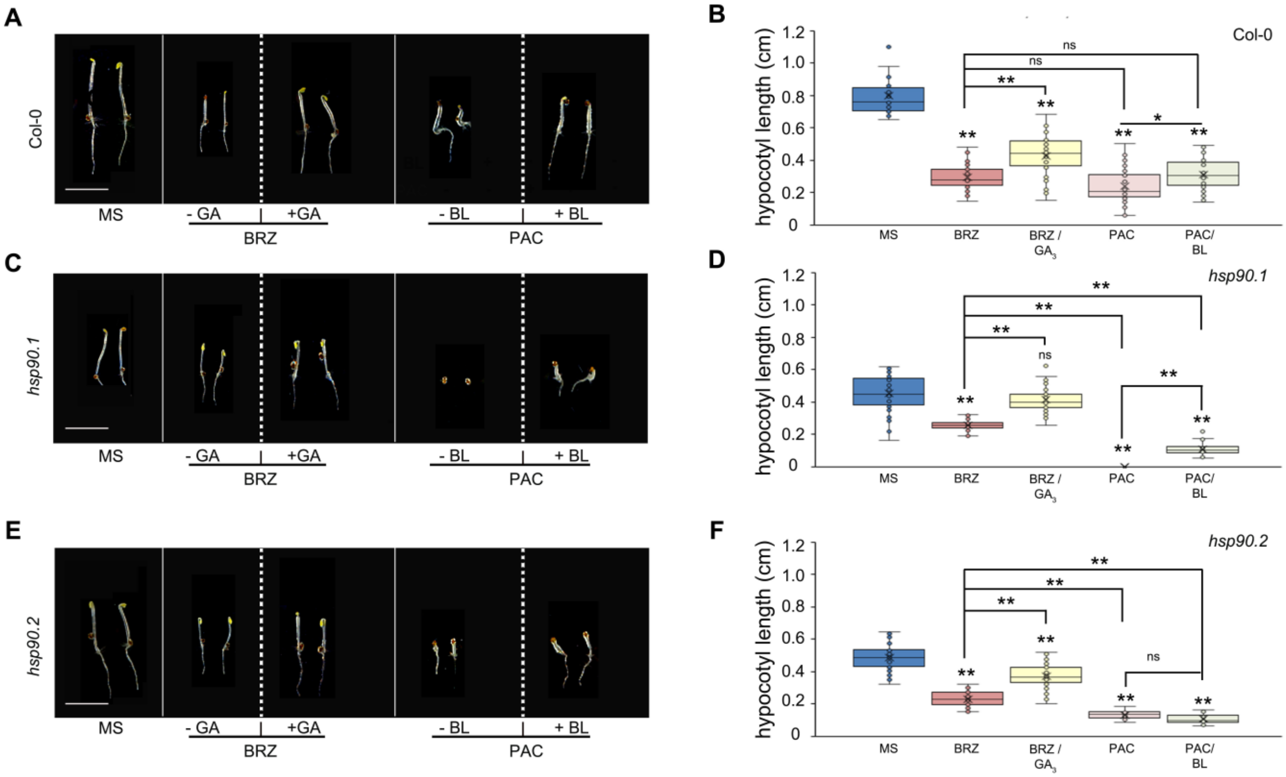

Figure S5. Hypocotyl elongation assays of wild type *hsp90* etiolated seedlings under deficiency of BRs or GAs. **(A, C, E)** Images of 5-day-old etiolated seedlings Col-0 (**A**), *hsp90.1* (**C**) and *hsp90.2* (**E**) growing in the indicated conditions. (**B, D, F**) Hypocotyl elongation of 5-day-old Col-0 (**B**), *hsp90.1* (**D**) and *hsp90.2* (**F**) growing on control MS medium, or in the presence of 0.5 µM BRZ, or in combination of 0.5 µM BRZ with 10 µM GA3 or in the presence of 0.1 µM PAC or in combination of 0.1 µM PAC with 60 nM BL. Box plots display the first and third quartiles, split by the median; whiskers extend to include the max/min values. Statistical analysis was performed using one-way analysis of variance (ANOVA) followed by Tukey test for multiple comparisons, ns: non-significant, * $p<0.05$, ** $p<0.01$.

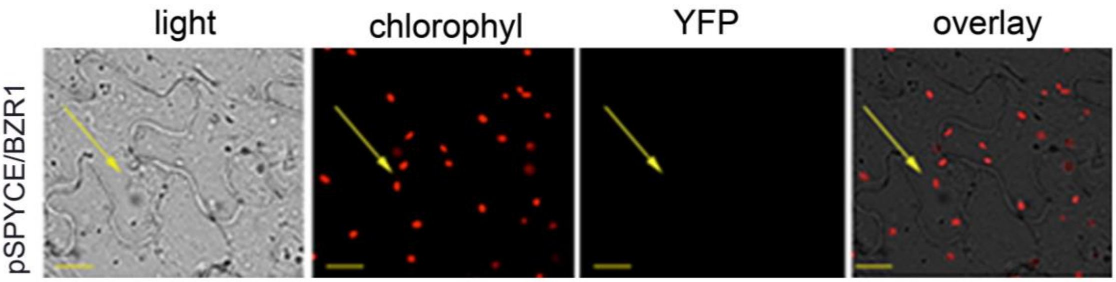

Figure S6. Negative control of HSP90.1 or HSP90.2 BiFC interaction assays with BZR1. *N. benthamiana* leaf epidermal cells co-transformed with pSPYCE-YFPc and BZR1-nYFP were used as controls. No fluorescence was detected. Arrows indicate the nucleus. Scale bars, 20 μm.

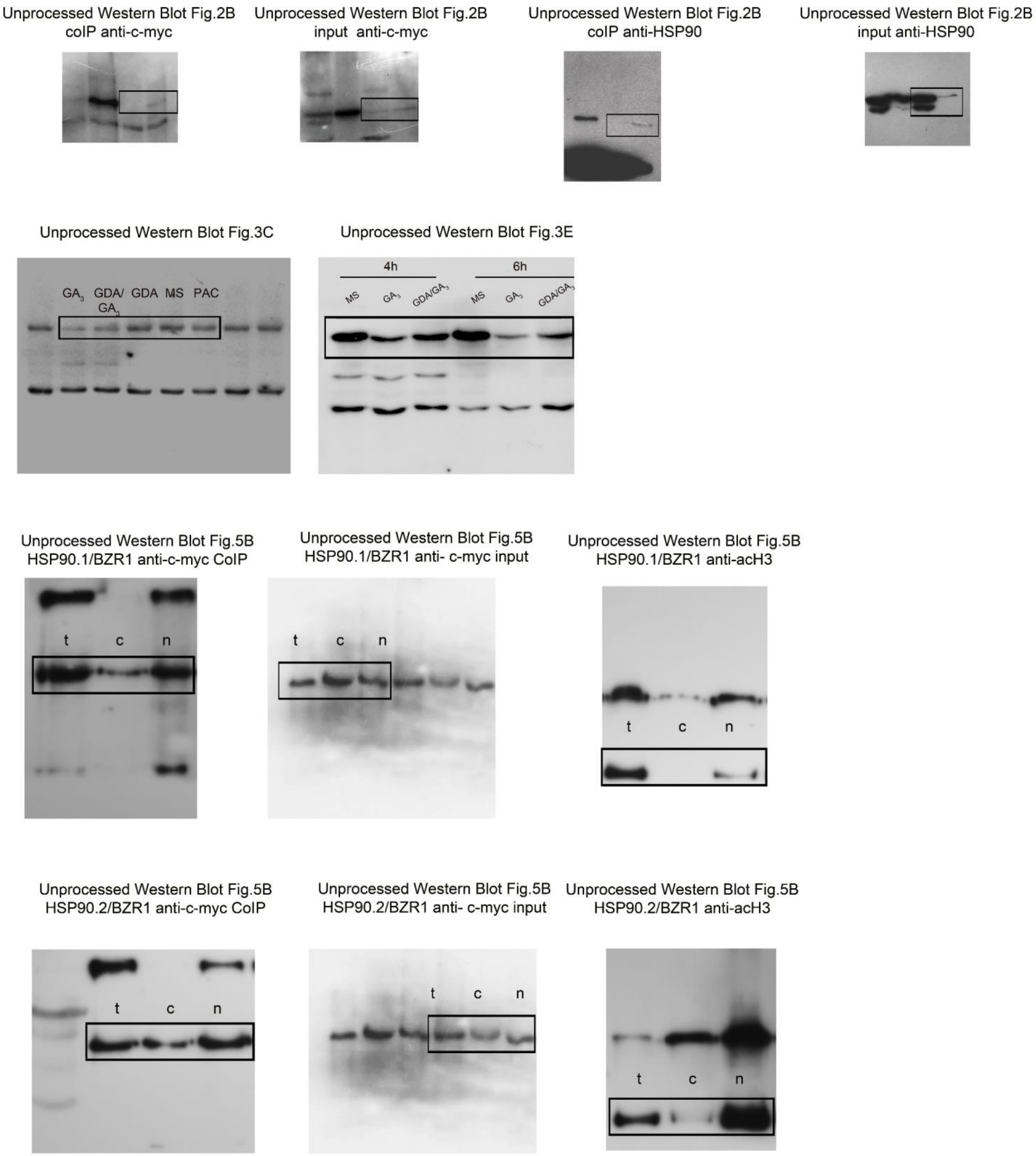

Figure S7. Unprocessed Western blot images used in this study.

Table S1. List of primers used in this study

Primers for yeast two hybrid interacrions		
Gene	Primer	Sequence
At5g52640	Hsp90.1yeastFor	5’-AAAGGATCCCGATGGCGGATGTTCAG-3′
	Hsp90.1yeastRev	5’–TTTCTCGAGGTCGACTTCCTCCATCTTGC-3′,
At5g56030	Hsp90.2yeastFor	5′-ATACATATGATGGCGGACGCTGAAACC-3′
	Hsp90.2yeastRev	5′-ATAATCGATTTAGTCGACTTTTGTGTG-3′
At1g75080	Bzr1yeastFor	5′-TTTTCATATGATGACTTCGGATGGAGCTACGTCG-3′
	Bzr1yeastRev	5′-AAAACCCGGGACCACGAGCCTTCCCA-3′
At2g01570	RGA1yeast For	5′-CCTCGGATCCGAATGAAGAGAGATCATCATCATCATC-3′
	RGA1yeast Rev	5′-ATTTGTCGACATTGGTGGAGAGTTTCCACGC-3′
At1g14920	GAI1yeast For	5′-ATATGGATCCGAATGAAGAGAGATCATCACCAATTCC-3′
	GAI1yeast Rev	5′-TTTTGTCGACGTACGCCGCCGTCGA-3′
Primers for BiFC contructs		
Gene	Primer	Sequence
At5g52640	Hsp90.1splitFor	5′-TTAATGGATCCAAGTTCGTTGCGATGGCGGATG-3′
	Hsp90.1splitRev	5′- TTTCTCGAGGTCGACTTCCTCCATCTTGC-3′
At5g56030	Hsp90.2splitFor	5′-ATATCTAGAACGACAATGGCGGACGCTGAAACC-3′
	Hsp90.2splitRev	5′-ATAGGTACCGTCGACTTCCTCCATCTT-3′
At1g75080	Bzr1splitFor	5′-ACTAGTATGACTTCGGATGGAGCTACG-3′
	Bzr1splitRev	5′-TTTTGGTACCACCACGAGCCTTCCCATTTCC-3′
At2g01570	RGA1split For	5′-CCTCGGATCCATGAAGAGAGATCATCATCATCATC-3′
	RGA1split Rev	5′-ATTTGTCGACATTGGTGGAGAGTTTCCACGC-3′
At1g14920	GAI1split For	5′-ATATGGATCCATGAAGAGAGATCATCACCAATTCC-3′
	GAI1split Rev	5′-TTTTGTCGACGTACGCCGCCGTCGA-3′
Primers for Quantitative RT PCR (qPCR)		
Gene	Primer	Sequence
	oligo -dT	5′-GTCGACCTCGAGTTTTTTTTTTTTTTTTT-3′
At1g02400	qGA2ox6 - F	5' -ATCCAACGACGTAGATGGACTTG-3′
	qGA2ox6 - R	5' -CCTATGCCTCACGCTAGTGAAT-3′
At1g05160	qKAO1 fwd	5' -TGAGGTTCTACAAAGAGCAAAGGC-3′
	qKAO1 rev	5' -ACTCGAAGTGTCTCATCGACAACC-3′
At4g25420	qGa20_ox1 fwd	5' -AGATTACTTCTGCGATGCGTTGG-3′
	qGa20_ox1 rev	5' -CGAATGGAGCGCCATTGATTTTC-3′
At1g30040	qGa2_ox2 fwd	5' -AGATGGAAGTTGGGTCGCTGTC-3′
	qGa2_ox2 rev	5' -TGTATCGGCTAAGACCCTGTG-3′
At1g15550	qGA3ox1 - F	5' -GTCTAGCAGCTCATACCGACTC-3′
	qGA3ox1 - R	5' -CCATTGGATAGGATGTGGAAGAGG-3′
At1g13440	qGAPDH - F	GCCAAGAAGGTTGTTATCTCTGCCC-3′
	qGAPDH - R	5' -GCTCGACCTGTTGTCGCCAACG-3′
At2g37640	qEXP3-F	5' -TCCATCTATTCTTGTGACGGCGAC-3′

	qEXP3-R	5' -TCCCTCCTATCTTCCGACAAGGTA-3′
At2g40610	qEXP8 F	5' -TCCTCCTCTTCAGCATTTCGACCT-3'
	qEXP8 R	5' -CTTGCCACGACTGTGTTTTTGAGC-3′
At4g30290	qXTH19-F	5' -GACCCAACCGCTAACTTTCACAC-3′
	qXTH19-R	5' -AGGAGCTTTTGACCAATCTGTTTTCTC-3′
At1g10550	qXTH33-F	5' -ATACCCTCATTTGGAACTCCCACC-3′
	qXTH33-R	5' -CACTTAACTCCACGTCAGCAACG-3′
